# The Impact of Hepatic Hydrothorax on the Outcome of Liver Cirrhosis: A Comparative Study

**DOI:** 10.3390/jcm14010212

**Published:** 2025-01-02

**Authors:** Sandica Bucurica, Ioana Parolă, Alexandru Gavril Vasile, Ionela Maniu, Mihaela-Raluca Mititelu

**Affiliations:** 1 Department of Gastroenterology, “Carol Davila” University of Medicine and Pharmacy Bucharest, 020021 Bucharest, Romania; sandica.bucurica@umfcd.ro; 2 Department of Gastroenterology, “Dr. Carol Davila” Central Military Emergency University Hospital, 010825 Bucharest, Romania; vasile_andi@yahoo.com; 3 Department of Gastroenterology, Fundeni Clinical Institute, 022328 Bucharest, Romania; ioana.prodan96@gmail.com; 4 Department of Mathematics and Informatics, Faculty of Sciences, Lucian Blaga University Sibiu, 550012 Sibiu, Romania; 5 Research Team, Pediatric Clinical Hospital Sibiu, 550166 Sibiu, Romania; 6 Department of Nuclear Medicine, University of Medicine and Pharmacy Carol Davila Bucharest Romania, 020021 Bucharest, Romania; raluca.mititelu@umfcd.ro; 7 Department of Nuclear Medicine, University Emergency Central Military Hospital, 010825 Bucharest, Romania

**Keywords:** hepatic hydrothorax, cirrhosis, pleural effusion, MELD, Child–Pugh–Turcotte

## Abstract

**Introduction:** Hepatic hydrothorax (HH) is a severe cirrhosis complication requiring early diagnosis and appropriate management. This study aimed to assess the impact of HH on the disease severity and mortality of cirrhotic patients and compare their clinical and biological profiles with those of patients without HH. **Materials and Methods:** This retrospective study involved 155 patients diagnosed with cirrhosis, of whom 31 had HH. The diagnosis of HH was based on imaging techniques such as X-ray, ultrasound, and thoracic CT scans. The severity of cirrhosis was evaluated using the Child-Pugh, MELD, MELD-Na, and MELD 3.0 scoring systems. **Results:** Of the included patients, 83.87% (*n* = 26) were men, with a 20% incidence of HH. The main etiology was chronic alcohol use. The pleural fluid localization revealed similar numbers of patients with bilateral and right pleural distribution. Patients with HH were predominantly classified in Child–Pugh–Turcotte class C. The MELD, MELD-Na, and MELD 3.0 scores had higher median values in the group of patients with hepatic hydrothorax. Still, death occurred at lower MELD scores when compared with cirrhotic patients without HH (MELD score > 22.5 for patients with HH vs. MELD > 32.5 for patients without HH). (The cirrhotic patients with HH presented lower serum albumin, cholesterol, and triglyceride levels and higher bilirubin, INR, and creatinine values. The mortality rate was higher in the group with HH-58,06% versus 20.97% in the control group (cirrhotics without HH). **Conclusions:** Hepatic hydrothorax is a serious complication of cirrhosis that requires early recognition and proper management, supported by using biomarkers and scoring systems.

## 1. Introduction

Cirrhosis can develop due to chronic liver disease caused by persistent liver injury and fibrosis. Portal hypertension and progressive liver dysfunction, as consequences of cirrhosis, imply various complications such as ascites, variceal bleeding, hepatic encephalopathy, coagulation disorders, hepatorenal syndrome, cirrhotic cardiomyopathy, sarcopenia, and hepatocellular carcinoma [1,2,3]. Pulmonary complications might include hepato-pulmonary syndrome (HPS), porto-pulmonary hypertension (PPHT), hepatic hydrothorax, or spontaneous bacterial empyema [4].

Hepatic hydrothorax, a relatively uncommon complication of cirrhosis, represents the presence of fluid in the pleural space, and it is associated with the end stages [5,6]. This is a consequence of portal hypertension, and it is not correlated with any other underlying cardiopulmonary disorder [7,8]. It becomes clinically evident when at least 500 mL of transudative fluid fills the pleural cavity [9,10,11,12]. In most cases, HH develops on the right side of the thorax. Still, it can also be found on the left side or bilaterally [13]. The fluid distribution on the right side is related to embryological development. The left hemidiaphragm has more muscular fibers, while the left side contains collagenous fibers [7,8,14].

**Figure 1:**
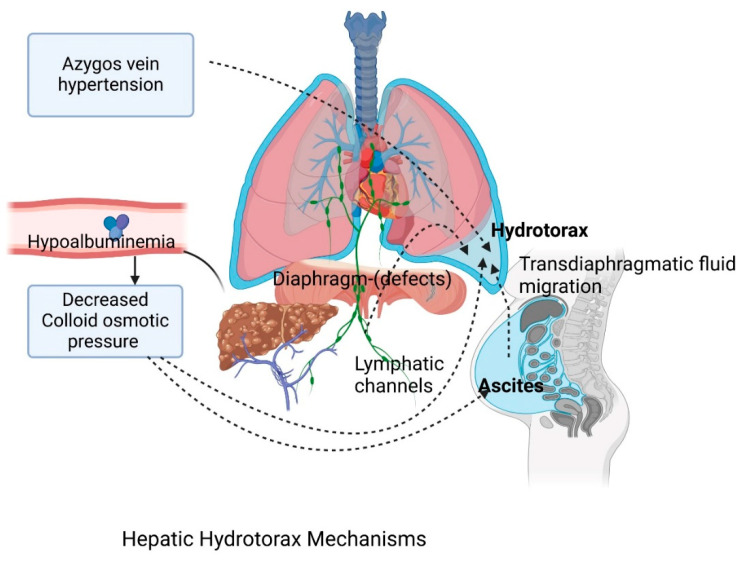
The proposed pathophysiological mechanisms of hepatic hydrothorax. There are various mechanisms responsible for hepatic hydrothorax appearance in cirrhotic patients: hypoalbuminemia, secondary to decreased colloid osmotic pressure, which can cause ascites and pleural effusion; increased pressure in azygos veins and trans-diaphragmatic migration of the fluid via the lymphatic vessels. Another mechanism that has been studied is the direct flow through diaphragmatic defects from the peritoneal cavity to the pleural space [16,17]. (Created in BioRender).

The exact mechanism is not fully understood but appears to have multiple causes. Decreased colloid pressure, secondary to hypoalbuminemia [15,16,17], increased pressure in azygous veins [16], the transdiaphragmatic migration of fluid into the pleural space through lymphatic vessels [16,17], and diaphragmatic defects that can cause direct passage of the fluid secondary to increased intraabdominal pressure (ascites, cough), which have been the most widely studied, have all been postulated [10,16,18,19,20,21] (Figure 1).

Usually, this condition is symptomatic; patients may experience shortness of breath, hypoxia, cough, fatigue, nausea, and signs of respiratory distress [14,22]. The diagnosis is made using frontal chest radiography, and to ensure the lack of other complications, a CT scan or a thoracentesis can follow [23]. Alongside pleural effusions, typically, there is a moderate to severe amount of ascitic liquid, and less often, there are cases with HH and no ascites [23,24]. Similarly to spontaneous bacterial peritonitis (neutrophils in ascitic fluid > 250/mm^3^), HH can be complicated by bacterial infection of the fluid into the pleural cavity, causing spontaneous bacterial empyema (SBEM) [23,25]. This condition has a high mortality rate, usually caused by Escherichia coli, Streptococcus species, and Klebsiella pneumoniae [23,25].

For HH’s adequate management, a multidisciplinary approach is required [21,26], from the implementation of a sodium-restricted diet, diuretics, and abstinence from alcohol-inducing factors to multiple therapeutic thoracenteses, chest tube insertion, and liver transplantation [14,26,27,28,29]. However, multiple thoracocenteses in cirrhotic patients with HH have been associated with a high risk for pneumothorax, hemothorax, hematoma, pulmonary edema, and other complications [14,20]. Hepatic hydrothorax is associated with high mortality and morbidity, with a median survival of 8–12 months [8].

This article aimed to evaluate different aspects of HH patients compared with patients suffering only from cirrhosis. Multiple parameters were used to establish how this complication influenced the mortality and morbidity rates, its association with end-stage liver conditions, the dynamics of various biomarkers, and the outcome of these patients.

## 2. Materials and Methods

**Figure 2:**
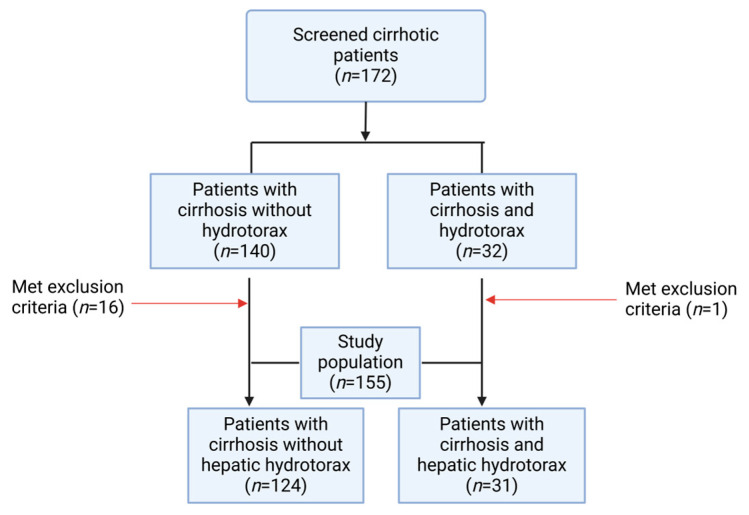
Study flow diagram.

This is a single-center retrospective study. All patients diagnosed with liver cirrhosis admitted between January 2020 and May 2024 to the University Emergency Central Military Hospital Bucharest were included and then were split into two groups—with and without hepatic hydrothorax. (Figure 2). The diagnosis of hepatic hydrothorax was based on the presence of pleural effusion on a chest X-ray, ultrasound, or thoracic CT scan. Decompensated cirrhosis has been defined according to the EASL clinical Practice Guidelines [30]. The severity of cirrhosis was evaluated using the Child-Pugh score and the MELD, MELD-Na, and MELD 3.0 scoring systems [18,31,32].

This cohort was split into two groups according to the presence or absence of HH to compare demographic, clinical, and biological parameters. A total of 155 cirrhotic patients were enrolled, 31 of whom had HH and 124 of whom did not have HH (Figure 2).

We excluded patients with cardiopulmonary conditions, those under 18 years old, patients who underwent TIPS or liver transplantation, those who had malignancies, those on immunosuppressive medications, and patients with insufficient clinical data.

This study was conducted in accordance with the Declaration of Helsinki and was approved by the Ethics Committee of Carol Davila Central Military Emergency University Hospital, Bucharest, no 740/01.11.2024. Informed consent was obtained from all patients.

We collected a series of data including the following: age, sex, etiology, the presence of ascites, hepatic encephalopathy, esophageal varices, the need for ICU admission, and biological profiles—leucocytes, platelets, total (TB) and direct bilirubin (DB), lactate dehydrogenase (LDH), albumin, alanine aminotransferase (ALT), aspartate aminotransferase (AST), alkaline phosphatase (ALP), gamma-glutamyl transpeptidase (GGT), urea, creatinine, sodium (Na), potassium (K), and the international normalized ratio (INR). The analysis of the pleural serum was performed according to the Light criteria for transudative effusion—total protein < 2.5 g/dL; pleural fluid/serum total protein ratio < 0.5; pleural fluid/serum LDH ratio < 0.6; and a gradient of serum albumin to pleural fluid (SPAG) > 1.1 [17,23].

All blood samples were analyzed in the clinical laboratory of “Carol Davila” Central Military Emergency University Hospital. The blood count and coagulation tests were performed with Sysmex XN3000 (Etten Leur, The Netherlands). For biochemistry evaluations (of albumin, total cholesterol, triglycerides, aspartate aminotransferase, alanine aminotransferase, alkaline phosphatase, gamma-glutamyl transpeptidase, total bilirubin, direct bilirubin, glycemia, urea and creatinine values), was used the Beckmann Coulter AU5822 analyzer (Beckman Coulter, Brea, CA, USA), along with the spectrophotometry method.

### Statistical Analysis

Continuous variables are reported as medians and interquartile range (IQR: percentile 25–percentile 75). Categorical data are expressed as numbers (n) and proportions (%). Comparisons between groups (with versus without hepatic hydrothorax) were performed using the Mann–Whitney U test for continuous variables and Chi-square or Fisher’s exact test for categorical variables. The Kaplan–Meyer curve was used for survival analysis. A logistic regression model and univariate and multivariate analyses were used to determine the independent mortality-related factors. A *p*-value < 0.05 was considered significant. Statistical analyses were performed using IBM SPSS version 20. We also used a non-linear, multivariate, supervised machine learning technique, the CART decision tree method, to assess the interactions/interrelations between different variables, cut-off points for them, and their relationship with death (patient risk groups).

## 3. Results

Of the 155 patients included, 83.87% (*n* = 26) males were in the group of patients with HH, and 66.13% (*n* = 82) males were in the control group. The mean age was 61.64 ± 10.74 for all patients with a younger age for the group of patients with hepatic hydrothorax (61 vs. 64 for the control group). The incidence of HH in our study was 20%. The predominant etiology was related to alcohol abuse (64.52% in the control group, 83.87% in the group with HH) followed by viral infection—21.77% in the control group vs. 9.68% for the patients with HH (10 patients with B ± D infection, 15 patients with C infection and 2 patients with B + C infection in the control group vs. 1 patient with B infection and 2 patients with C infection in the group of patients with HH) (Table 1).

Hepatic hydrothorax occurred in 3.23% of patients with Child–Pugh–Turcotte class A and 12.90% with class B; the majority were in the Child–Pugh–Turcotte class C—83.87%. The MELD, MELD-Na and MELD 3.0 score revealed higher median values in the group with HH (19 vs. 15 for MELD score, *p* = 0.003; 23 vs. 16 for MELD-Na score, *p* = 0.000; 22 vs. 16, *p* = 0.001 for MELD 3.0 score, *p* = 0.001).

Among the cases with HH, the same number of patients had bilateral and right pleural effusions—38.71% (*n* = 12) (while the cirrhotic patients with left pleural effusions were 22.58% (*n*= 7). Paracentesis was performed on 70.97% of the cirrhotic patients with HH, while thoracocentesis was conducted only in 54.84% (*n* = 17) of the patients. Among these, 45.16% (*n* = 14) had their pleural serum analysis evaluated according to Light criteria, with 78.57% (*n* = 11) showing a transudate. Additionally, after calculating the pleural albumin gradient (SPAG), all had an SPAG over 1.1 g/dL, confirming the transudative appearance in all patients. Moreover, 11.76% (*n* = 2) of all patients with HH presented SBEM.

The comparison of the biological parameters revealed higher INR values for the patients with HH, in a range of 1.38–2.11, higher than those in the group of patients without HH. Moreover, the observed group’s creatinine and urea levels were slightly elevated. Serum albumin levels were lower in the group with HH and ranged from 2.05 to 2.70 g/dL. The hepatic enzymes had similar values in both groups, but the bilirubin level in the cirrhotic patients with HH was almost double that of participants in the control group. The lipid profile revealed lower levels of cholesterol and triglycerides in the HH- cirrhosis group compared with the values found in the control group. The glucose levels were similar in both groups, but the hemogram showed a higher level of leukocytes in the group of patients with HH, ranging from 5.33 to 15.66 k/mL. The platelet count was lower in cirrhotic patients without HH. Both groups presented a median of serum sodium levels lower than the normal range, but cirrhotic patients with HH had a wider variance interval.

An association between leucocytes, cholesterol, bilirubin, INR, Na, and albumin levels was found (Table 2).

The mortality rate was higher in the group of patients with hepatic hydrothorax—58.06% (*n* = 18)—than in the control group—20.97% (*n* = 26). Moreover, 48.39% (*n* = 15) of the patients with HH needed ICU admission (Table 1).

**Figure 3:**
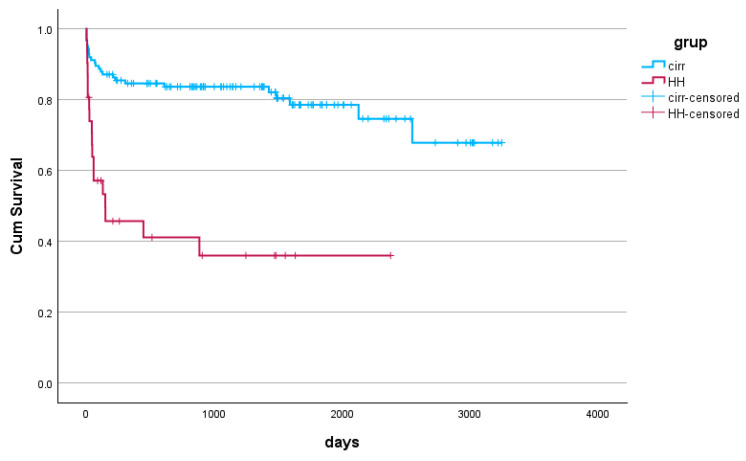
The survival probability of the two groups: cirrhotic patients with and without hepatic hydrothorax (*p* < 0.001).

The Kaplan–Meier survival analysis revealed a steeper slope for the group with HH in the first 1000 days, indicative of a higher mortality rate early on but with a flatter aspect after, associated with a 50% survival rate. The cirrhotic patients without HH had a flatter slope from the beginning, with a slight decrease after 2000 days, showing a gradual decline in survival. The analysis indicates that HH significantly impacts early survival, but patients who survive this critical phase have a slightly better outlook (Figure 3).

The 30-day, 60-day, and 120-day mortalities in the group of patients with HH were 29.03%, 45.16%, and 51.61%, respectively.

Univariate analysis revealed risk factors related to death, such as hepatic hydro-thorax (OR: 5.219, 95%CI: 2.266–12.019), ascites (OR: 5.205, 95%CI: 1.733–15.639), encephalopathy (OR: 6.994, 95%CI: 3.213–15.225), Child-Pugh-Turcotte C class (OR: 13.404, 95%CI: 3.022–59.453), MELD (OR: 1.184, 95%CI: 1.144–1.258), and MELD-Na (OR: 1.150, 95%CI: 1.090–1.212). In the multivariate analysis, hepatic hydrothorax (OR: 5.584, 95%CI: 1.623–19.210), encephalopathy (OR: 5.433, 95%CI: 1.913–15.425), and MELD (OR: 1.251, 95%CI: 1.051–1.489) were independent risk factors for death (Table 3).

**Figure 4:**
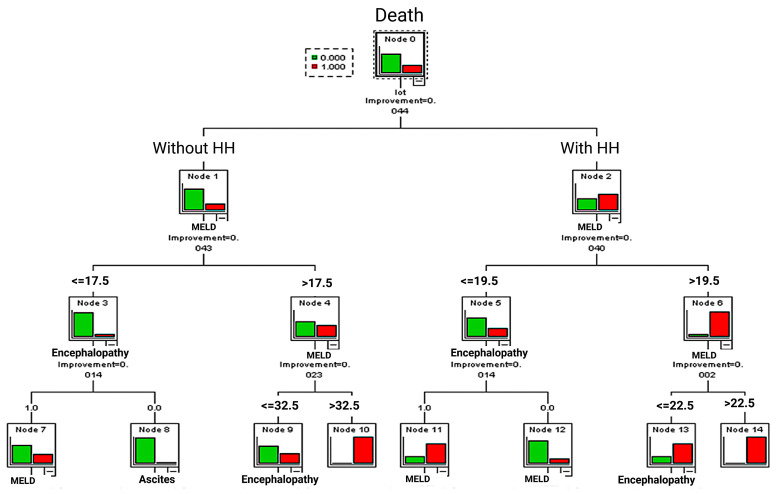
CART decision tree method.

The results of the CART decision tree method showed that patients with HH presented death at a lower MELD score (MELD > 22.5) (Node 14: death in 10 cases (100%)). In the case of patients without HH, death was observed in all patients with a MELD score > 32.5 (Node 10: death in five cases (100%)) (Figure 4).

Death occurrence in cases with hepatic hydrothorax was observed in 100% of patients with a MELD score > 22.5. Inpatients without hepatic hydrothorax death was associated with a MELD score > 32.5. (red column—deceased patients; green column—survivors)

In the case of a MELD score < 19.5 for patients with HH and a MELD score < 17.5 for patients without HH, it was observed the presence of encephalopathy(Node 11, Node 7). (HH—hepatic hydrothorax) (Figure 4).

## 4. Discussion

Comparing our results (20% cases with HH) with the published ones, the prevalence of HH was slightly higher than that in the reported data [5,24,33]. Badillo et al. reported that 16% of the included patients developed HH, while Matei et al. reported a 13.1% prevalence of HH in the study group.

The predominant etiology in our study was related to alcohol use. The report from the World Health Organization in 2019 for Romania emphasized that the prevalence of heavy alcohol drinking is 34.8%, which is higher than the mean for Europe (30.4%), and that 71% of alcohol drinkers might develop cirrhosis [34]. Moreover, Romania is in ninth place on the list of heavy drinkers from European countries [35]. Hepatitis B virus infection is the most prevalent etiology seen in published articles related to HH [33,36,37,38], still a study evaluating the Romanian population found that alcohol abuse is the most frequent burden [5]. Regarding hepatitis C virus infection, the reduced prevalence in this study may be related to the use of direct-acting antivirals (DAAs) for eradication and different programs conducted in Romania, including rural areas and prisons [39,40,41,42].

Abassi evaluated the influence of the Child–Pugh class on cirrhotic patients with HH in 2016. He identified that 65% of the patients had Child–Pugh class C, 26% were in the B class, and 9% were in the A class [43]. Chen et al. (2020) found that 8.3% of the patients had Child–Pugh class A, 16.7% were in the B class, and 75% were in the C class [36] and a Romanian study found that more than half of the patients with HH (*n* = 100) were in the Child–Pugh C class [5]. In our study, 83.87% of the patients with HH were in the Child–Pugh C class. Moreover, the MELD, MELD-Na, and MELD 3.0 scores were higher in the HH group, similar to data documented in previous publications [5]. Even though the MELD and MELD-Na scores are reliable scores used for liver transplant evaluation, MELD-Na is an inconsistent tool for predicting survival in cirrhotic patients with HH [5]. The MELD 3.0 score has shown improved accuracy in predicting mortality related to hepatic causes for cirrhotic patients with HH [9]. Moreover, in patients who needed pleural effusion aspiration, it was found that there was a higher risk of acute-on-chronic liver failure or death [44]. Furthermore, a study evaluating patients with cirrhosis and HH on the liver transplant waiting list found that those receiving MELD exception points for HH had a reduced post-transplant mortality rate compared with those who did not (OR 0.56; 95% Cl 0.37–0.88; *p* = 0.01) [45].

The appearance of hepatic hydrothorax without ascites has already been postulated and can even be the initial indicator of cirrhosis [22,38,46,47]. In our research, few patients exhibited HH without concurrent ascites.

There are limited studies evaluating the biological profile of cirrhotic patients with HH. It has been found that patients with HH have lower cholesterol and triglyceride levels [33], and that their hepatic insufficiency is more advanced, exhibiting higher INR values [5,33,38], lower serum albumin levels [5,37,38], and higher bilirubin values [5,37,38,48]. Moreover, congruently with our study’s results, they tend to express higher creatinine levels [5,37,38]. Lower sodium levels in patients with HH have been uncovered in some reports, and our results are consistent with this finding [37,49]. Moreover, hyponatremia (<130 mmol/L) proved to be a prognostic factor for hepatic hydrothorax (OR. 5.723, Cl 1.889–17.336; *p* = 002) [50].

Besides modified laboratory markers, diabetes, recurrent paracentesis, and non-use of non-selective beta-blockers were found to be risk factors for HH development [48]. The last two have not been evaluated in this research, and diabetes was not correlated with hepatic hydrothorax in our study.

Even though the exact pathophysiological mechanism of hepatic hydrothorax is not fully understood, it has been explained through the years by introducing CO2 into the abdomen of patients with HH and revealing a pneumothorax using X-rays after 48 h or injecting blue methylene into the peritoneal cavity. Furthermore, scintigraphy with [99 Tcm]-human albumin or [99 Tcm]-sulfur colloid has shown a unidirectional passage from the abdomen to the pleural cavity [10,23,51,52]. Several studies have tried to find a connection between the development time and the role of pleural effusion in cirrhotic patients. It has been emphasized that HH localization has no connection with the severity of cirrhosis. Bilateral pleural effusion is higher in cirrhotic patients with portal vein thrombosis, which has been linked to increased pressure in the umbilical and hemiazygos veins [33].

Another consequence of portal hypertension is hepato-pulmonary syndrome (HPS). At first, a connection between HH and HPS was proposed, but studies have proven the differences between these two entities over time [4,53]. HPS is a result of excessive vasodilatation into the pulmonary vasculature caused by numerous molecules—nitrogen (NO), carbon monoxide (CO), and endothelin-1 (ET1)—and monocyte overstimulation, which predisposes individuals to vascular endothelial growth factor (VEGF) activation and neo-angiogenesis. In addition, portal hypertension determines bacterial translocation, a major contributor to further vasodilators released into the pulmonary vascular endothelium [4,53].

The treatment options for hepatic hydrothorax comprise a low-sodium diet and diuretics, while for symptomatic patients with large pleural effusion, thoracocentesis might be necessary [8,21,54]. In our study, half of the patients with hepatic hydrothorax underwent thoracocentesis, and 45.16% had their pleural serum evaluated, revealing a transudate.

To avoid the confusion with an exudate pleural effusion, in patients under diuretics was recommended to use serum-pleural fluid albumin gradient (SPAG) [23]. Furthermore, the incidence of SBEM of 11.76%- that we found in our study, is comparable with the results reported in the literature [55,56].

Regarding the treatment possibilities for HH, research has assessed the effectiveness of intravenous terlipressin, octreotide, or adding midodrine, an alpha-adrenergic agonist, to octreotide for managing HH. These studies indicate a potential benefit in reducing pleural fluid accumulation due to decreased splanchnic blood flow, but additional data are needed [57,58,59,60]. Moreover, it has been emphasized that paracentesis should be attempted for cirrhotic patients with HH before thoracocentesis to prevent rapid fluid accumulation [57].

Hepatic hydrothorax has a significant influence on the survival of patients. Deleuran et al. found that HH has a high mortality hazard ratio of −4.35 (Cl 2.76–6.97) [48]. Moreover, the mean survival for cirrhotic patients with hepatic hydrothorax was 4.73 months (range: 0.03–60 months) in a Taiwanese study [36], and O’Leary et al. reported a 4.78 monthly survival rate with a 51.06% one-year mortality rate in 2021 [44]. The short-term mortality rate at 30 days, 90 days, and 1 year reported by Hung was 20.1%, 40.2%, and 59.1% for cirrhotic patients with HH [27], while another study found a mortality rate of 50.5% in one year for the same group [5]. Congruently with the literature data, our study found relatively similar rates of 30-day, 60-day, and 120-day mortalities (29.03%, 45.16%, and 51.61%) in the group of patients with HH.

Recently published data showed that a promising scoring system (The CIRrhotic Ascites Severity—CIRAS) was used to predict the appearance of HH in cirrhotic patients (*n* = 1198) and proved to help evaluate HH development (AUROC-0.67) [61].

One limitation of this retrospective single-center study is the small cohort. Another limitation is the lack of therapy evaluation on the HH groups, the lack of assessment of the ascites and pleural effusion grades, and the short period of monitoring. The strength of our study is that it is one of the few regarding the outcome of hepatic hydrothorax in a Romanian cohort and presents significant comparative results.

## 5. Conclusions

Hepatic hydrothorax has been continuously evaluated in recent years, and more data have appeared regarding its pathological mechanisms, the evolution of this complication, the relationship to other potential complications of cirrhosis, and the treatment options, including TIPS and liver transplantation. Hepatic hydrothorax has been shown to alter the evolution of cirrhosis, interfering with its pathological mechanisms and leading to an increase in disease severity and mortality. Hepatic hydrothorax remains a significant complication of cirrhosis, unproperly evaluated, that should be diagnosed early and treated adequately and rapidly using biomarkers and scoring systems.

## Figures and Tables

**Table 1:** The comparison between the two groups: with and without hepatic hydrothorax.

Variables	Cirrhosis Without Hepatic Hydrothorax	Cirrhosis with Hepatic Hydrothorax	*p*-Value
Sex (N, %)—Male	82 (66.13)	26 (83.87)	0.055
Age (years)	64 (55–70)	61 (52–65)	0.132
Alcohol use (N, %)	80 (64.52)	26 (83.87)	0.192
Viral (N, %)	27 (21.77)	3 (9.68)	
Mixed (viral and alcohol) (N, %)	12 (9.68)	2 (6.45)	
Autoimmune (N, %)	5 (4.03)	0 (0.00)	
Diabetes mellitus (N, %)	39 (31.45)	6 (19.35)	0.184
Ascites (N, %)	84 (67.74)	29 (93.55)	0.004 *
Hepatic encephalopathy (N, %)	32 (25.81)	13 (41.94)	0.077
Esophageal Varices (N, %)	97 (78.23)	24 (82.76)	0.589
Child–Pugh–Turcotte A class (N, %)	36 (29.03)	1 (3.23)	0.001 *
Child–Pugh–Turcotte B class (N, %)	31 (25.00)	4 (12.90)	
Child–Pugh–Turcotte C class (N, %)	57 (45.97)	26 (83.87)	
MELD (N, IQR)	15 (10–21)	19 (15–28)	0.003 *
MELD-Na (N, IQR)	16 (10–24)	23 (20–31)	0.000 *
MELD 3.0 (N, IQR)	16 (6–38)	22 (9–43)	0.001 *
Intensive Care Unit (N, %)	19 (15.32)	15 (48.39)	0.000 *
Death	26 (20.97)	18 (58.06)	0.000 *

IQR-interquartile range, N-number, * *p* < 0.05.

**Table 2:** The biological comparison of the two groups.

Serum Parameters	Cirrhosis Without Hepatic Hydrothorax (Median, IQR)	Cirrhosis + Hepatic Hydrothorax (Median, IQR)	*p*-Value
Glucose level (mg/dL)	112.00 (93.00–143.00)	114.00 (87.00–137.00)	0.366
Leucocytes (k/microL)	6.63 (4.27–10.60)	8.49 (5.33–15.66)	0.037 *
Thrombocytes (k/microL)	136.00 (91.50–189.50)	154.00 (90.00–197.00)	0.739
Cholesterol (mg/dL)	127.00 (100.00–155.00)	81.50 (67.00–119.00)	0.003 *
Triglycerides (mg/dL)	91.00 (68.00–126.00)	82.50 (50.00–122.00)	0.311
AFP (ng/mL)	3.33 (2.51–5.65)	3.50 (2.07–4.92)	0.633
ALT (U/L)	24.00 (17.00–44.50)	25.00 (18.00–51.00)	0.516
AST (U/L)	45.50 (28.00–95.00)	57.00 (34.00–88.00)	0.342
GGT (U/L)	66.00 (37.20–119.00)	51.00 (22.70–134.70)	0.382
ALP (U/L)	98.00 (74.00–136.00)	103.50 (87.50–216.50)	0.125
Urea (mg/dL)	41.30 (26.30–64.00)	56.70 (29.70–118.80)	0.100
Creatinine (mg/dL)	0.79 (0.63–1.08)	1.05 (0.59–1.53)	0.251
TB (mg/dL)	1.50 (0.89–3.09)	3.64 (1.89–5.57)	0.000 *
DB (mg/dL)	0.48 (0.23–1.16)	1.56 (0.76–1.94)	0.000 *
INR	1.48 (1.23–1.75)	1.69 (1.38–2.11)	0.002 *
Na (mmol/L)	134 (116.00–141.00)	131 (109.90–142.00)	0.037 *
K (mmol/L)	4.21 (3.74–4.53)	4.25 (3.33–5.00)	0.955
Albumin (g/dL)	2.98 (2.40–3.78)	2.48 (2.05–2.70)	0.000 *

INR—international normalized ratio; AST—aspartate aminotransferase; ALT—Alanine aminotransferase; IQR—interquartile range; Na- serum sodium, K—serum potassium; TB—total bilirubin; DB— direct bilirubin; GGT gamma—glutamyl transferase-; ALP—alkaline phosphatase; AFP—Alpha-fetoprotein, * *p* < 0.05

**Table 3:** Univariate and multivariate analysis for mortality.

Characteristics	Univariate Analysis		Multivariate Analysis	
	OR (95% CI)	*p*-Value	OR (95% CI)	*p*
Sex	1.103 (0.519;2.344)	0.799		
Age	1.010 (0.978;1.044)	0.544		
Hepatic hydrothorax	5.219 (2.266;12.019)	0.000	5.584 (1.623;19.210)	0.006
Diabetes mellitus	0.755 (0.342;1.668)	0.487		
Ascites	5.205 (1.733;15.639)	0.003		
Hepatic encephalopathy	6.994 (3.213;15.225)	0.000	5.433 (1.913;15.425)	0.001
Esophageal Varices	1.909 (0.725;5.026)	0.191		
Child–Pugh–Turcotte				
B class	3.621 (0.379;19.316)	0.132		
C class	13.404 (3.022;59.453)	0.001		
MELD	1.184 (1.144;1.258)	0.000	1.251 (1.051;1.489)	0.012
MELD-Na	1.150 (1.090;1.212)	0.000		

## Data Availability

Data are available upon reasonable request due to privacy restrictions.
